# Applied Compressive Strain Governs Hyaline-like Cartilage versus Fibrocartilage-like ECM Produced within Hydrogel Constructs

**DOI:** 10.3390/ijms24087410

**Published:** 2023-04-18

**Authors:** Hamed Alizadeh Sardroud, Xiongbiao Chen, B. Frank Eames

**Affiliations:** 1 Division of Biomedical Engineering, College of Engineering, University of Saskatchewan, Saskatoon, SK S7N 5A9, Canada; 2 Department of Mechanical Engineering, College of Engineering, University of Saskatchewan, Saskatoon, SK S7N 5A9, Canada; 3 Department of Anatomy, Physiology, and Pharmacology, University of Saskatchewan, Saskatoon, SK S7N 5E5, Canada

**Keywords:** compressive force, hydrogel, hyaline cartilage, fibrocartilage, Col1, Col2

## Abstract

The goal of cartilage tissue engineering (CTE) is to regenerate new hyaline cartilage in joints and treat osteoarthritis (OA) using cell-impregnated hydrogel constructs. However, the production of an extracellular matrix (ECM) made of fibrocartilage is a potential outcome within hydrogel constructs when in vivo. Unfortunately, this fibrocartilage ECM has inferior biological and mechanical properties when compared to native hyaline cartilage. It was hypothesized that compressive forces stimulate fibrocartilage development by increasing production of collagen type 1 (Col1), an ECM protein found in fibrocartilage. To test the hypothesis, 3-dimensional (3D)-bioprinted hydrogel constructs were fabricated from alginate hydrogel impregnated with ATDC5 cells (a chondrogenic cell line). A bioreactor was used to simulate different in vivo joint movements by varying the magnitude of compressive strains and compare them with a control group that was not loaded. Chondrogenic differentiation of the cells in loaded and unloaded conditions was confirmed by deposition of cartilage specific molecules including glycosaminoglycans (GAGs) and collagen type 2 (Col2). By performing biochemical assays, the production of GAGs and total collagen was also confirmed, and their contents were quantitated in unloaded and loaded conditions. Furthermore, Col1 vs. Col2 depositions were assessed at different compressive strains, and hyaline-like cartilage vs. fibrocartilage-like ECM production was analyzed to investigate how applied compressive strain affects the type of cartilage formed. These assessments showed that fibrocartilage-like ECM production tended to reduce with increasing compressive strain, though its production peaked at a higher compressive strain. According to these results, the magnitude of applied compressive strain governs the production of hyaline-like cartilage vs. fibrocartilage-like ECM and a high compressive strain stimulates fibrocartilage-like ECM formation rather than hyaline cartilage, which needs to be addressed by CTE approaches.

## 1. Introduction

The knee joint cartilage undergoes different mechanical forces such as compression, tension, and shear during joint movements [1]. Cartilage tissue engineering (CTE) utilizes bioreactors to simulate mechanical conditions of the joints and to conduct animal experiments, since surgeries are expensive and challenging to perform [2,3]. However, it is not feasible to simulate the complex combination of several mechanical forces occurring in joints, and so, many bioreactors focused on simulating a single force. In this regard, dynamic compression is a primary type of force in the knee joint and several studies investigated how compression affects cartilaginous molecule biosynthesis in bioreactor environments [4,5]. Using bioreactors helps speed up the transition between in vitro testing and human trials by allowing constructs to be examined and modified before undergoing in vivo implantation [6].

Cell-impregnated hydrogel constructs are used extensively for CTE approaches in vitro and in vivo to regenerate articular cartilage [7,8]. Cells grow, differentiate, and maintain their chondrogenic morphology and phenotype in vitro in these hydrated networks with high levels of water, just like articular cartilage [9,10]. Despite several benefits of hydrogels for CTE, their structural characteristics and low mechanical properties should be taken into consideration for mechanically loaded in vitro bioreactors and in vivo joint implantations. Hydrogels easily transmit applied mechanical forces to the cells impregnated within them. The cells sense these forces through different mechanotransduction pathways and respond to them by deposition of different molecules [11,12]. Dynamic compression of chondrocytes impregnated in hydrogels-enhanced cartilage gene expression and matrix synthesis by formation of glycosaminoglycans (GAGs) and collagen type 2 (Col2) [13,14]. These molecules are major components of hyaline cartilage, which is also known as articular cartilage in the joints [15]. Collagen type 1 (Col1) is another molecule that cells may produce while differentiating and its production leads to a fibrocartilage-like extracellular matrix (ECM) formation which has inferior biological and mechanical properties compared with the native hyaline cartilage [16,17,18]. Most studies have investigated the formation of GAGs, total collagen, or Col2 from the constructs subjected to mechanical forces in vitro and in vivo [2]. Studies with a focus on Col1 production are limited, but those few studies that did assay for Col1 reported increased Col1 production in hydrogel constructs subjected to mechanical loading in vitro and in vivo [18,19,20].

Hence, this study hypothesized that mechanical compression stimulates fibrocartilage-like ECM production in hydrogel constructs. To test the hypothesis, hydrogel constructs were subjected to compressive forces and Col1 vs. Col2 deposition was assessed. The novelty of this study lies in the fact that it not only investigated the type of cartilage ECM produced in hydrogel constructs under mechanical compression, but it also applied varying magnitudes of compressive strains to mimic different activities that occur in vivo, such as walking, running, and jumping. For impregnating the cells, alginate hydrogel was used, as it is a proven biomaterial capable of stimulating cartilage matrix formation and chondrogenic differentiation of the cells [21,22]. The three-dimensional (3D)-bioprinting technique utilized in this study is a powerful tool in the current bioengineering industry due to its ability to design and fabricate complex architectures with an even distribution of cells at different regions of the constructs [23,24,25]. The cartilage in the joints undergoes around 10% compressive strain in vivo during walking; however, there are limited data about compressive strains caused by other activities such as running and jumping [26]. Accordingly, the fabricated hydrogel constructs were subjected to varying magnitudes of compressions from 6% to 24% using a bioreactor in efforts to mimic a variety of activities such as walking, running, and jumping [27]. The amounts of Col1 vs. Col2 deposited in the hydrogel matrix were determined using immunofluorescence staining for assessment of hyaline-like cartilage and fibrocartilage-like ECM productions. Additionally, histological staining and biochemical assessments were performed to confirm cell differentiation and cartilage matrix formation. The results revealed that compressive strain had a decisive role on the formation of hyaline-like cartilage vs. fibrocartilage-like ECM, and a high compressive strain stimulated the formation of fibrocartilage-like ECM rather than hyaline cartilage.

## 2. Results

### 2.1. Higher Strain Compression Damages the Constructs

**Figure 1:**
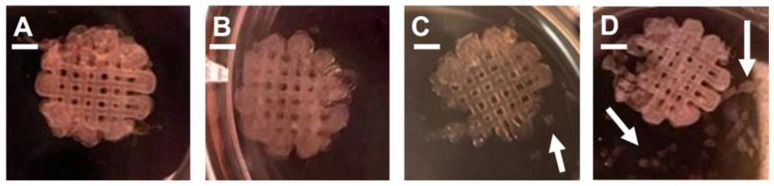
Representative images of (**A**) an unloaded construct as well as (**B**) 6%, (**C**) 12%, and (**D**) 24% loaded samples. The cleaved strands from the constructs following compression are indicated by arrows (Scale bar = 2 mm).

Based on macroscopic observations, unloaded and 6% loaded samples did not show clear changes in height and diameter (Figure 1A,B). However, other loaded groups had less height compared to their initial condition. Additionally, no structural changes were macroscopically visible for the unloaded and 6% loaded constructs (Figure 1A,B), but some regions of the 12% and more in 24 %loaded constructs experienced visible structural changes (Figure 1C,D). For example, incomplete strands were seen at the edges of these constructs and cleaved strands could be clearly observed inside the 6-well plates especially around the 24% loaded constructs (white arrows in Figure 1C,D).

### 2.2. Chondrogenic Differentiation of Cells Confirmed by Deposition of GAGs

**Figure 2:**
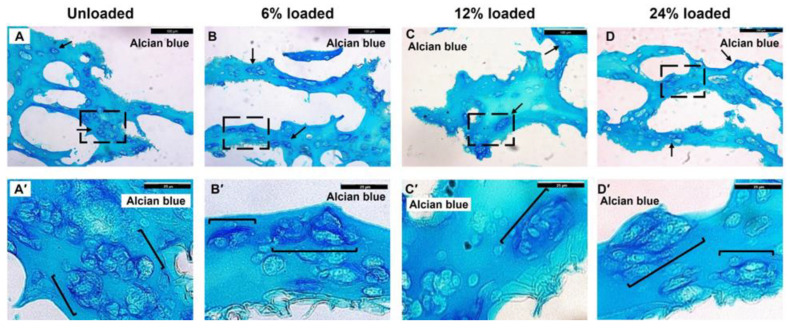
(**A**–**D**) Alcian blue stained sections show deposition of GAGs within different groups at various regions of the sections. (**A′**–**D′**) Higher magnification images of the dashed rectangles within the images of (**A**–**D**) show blue color related to GAG deposition surrounding the cells which is darker than the background blue color. The arrows and brackets are pointing to the cells depositing GAG (Scale bars: (**A**–**D**) = 100 µm, and higher magnification images of (**A′**–**D′**) = 25 µm).

Alcian blue staining was performed on the sections generated from the loaded and unloaded constructs to observe deposition of GAGs for chondrogenic differentiation of the cells. There was a varying level of background staining of the alginate hydrogel matrix seen in the sections (Figure 2), which was consistent with the literature on the subject [28,29,30]. Clusters of cells were seen in the images demonstrating that the cells had proliferated within the constructs. Lower magnification images of the sections showed that there were clusters of cells in outer peripheral and internal strands of the constructs (Figure 2A–D). In this manner, 3D-bioprinting proved beneficial in that it could provide enough pores for nutrients to diffuse into and waste to flow out of constructs, promoting a healthy environment for the proliferation of cells and differentiation of cell types. A darker blue color surrounding either single cells or clusters of cells indicated chondrogenic differentiation, distinguishing the deposited GAG from the lighter blue color of the background (Figure 2A′–D′, indicated with brackets). Additionally, clusters of cells did not join during the culture period as alginate matrix could be seen between the clusters (Figure 2). Previously, alginate hydrogels prepared at high concentrations were described as having a similar effect, trapping proliferated cells and deposited ECM and preventing the formation of homogeneous tissue-like structure [31,32].

### 2.3. GAGs and Collagens Tended Lower at Intermediate Strain Compression

**Figure 3:**
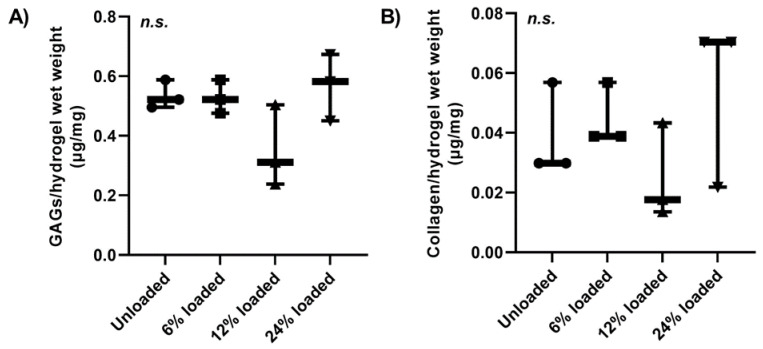
(**A**) Although there was no statistically significant difference in GAG deposition among the groups, the 12% group had the lowest median value, and (**B**) collagen contents of all groups also tended to be similar with slight variations between them that none were statistically significant. The bar graphs show median and interquartile range (n = 3 for each group, n.s. = not significant).

The levels of produced GAG in different unloaded and loaded constructs were quantitatively determined by performing DMMB assay as an assessment for chondrogenic differentiation of the cells. GAG production seemed to be similar for both unloaded (535 ± 27.5 μg/mg) and 6% loaded (529 ± 32.5 μg/mg) groups, and its production tended to decrease at 12% loaded group to 351 ± 79.5 μg/mg (Figure 3A). Further increase in compressive strain to a 24% compressive strain enhanced GAG production to a slightly higher level (568 ± 64.7 μg/mg) compared to the unloaded group. However, none of these changes were statistically significant (Figure 3A).

Total produced collagen within the constructs was also quantitated indirectly by measuring hydroxyproline content of the constructs. As with the GAG measurements, collagen production within the groups showed a similar trend, except that the unloaded group seemed to produce less collagen than the 6% loaded group (Figure 3B). Collagen production tended to decrease to 24.8 ± 9.3 μg/mg at the 12% loaded group, which appeared to have the lowest amount of produced collagen. It also seemed that the 24% loaded group produced the most collagen of all four groups (54.2 ± 16.2 μg/mg) with a mean of 40% more than the unloaded group. However, the SEM was large for the 24% loaded group, and none of the changes between groups were statistically significant (Figure 3B). 

### 2.4. Compressive Strain Is a Decisive Factor on the Produced ECM Type

**Figure 4:**
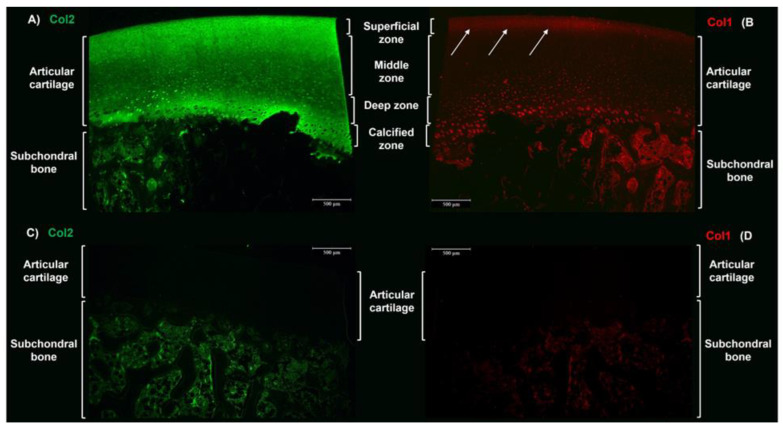
(**A**,**B**) Col1 and Col2 immunofluorescence staining on a same section of pig joint, as a positive control for the antibodies, confirmed that the selected primary antibodies detected Col1 and Col2 deposition at the expected regions, and arrows are pointing Col1 staining in the superficial zone. (**C**,**D**) Negative control section of a pig joint double immunofluorescence stained for Col1 and Col2 in the absence of the primary antibodies did not show any positive staining at the cartilage region (scale bars = 500 µm).

Immunofluorescence staining was performed to find the type and amounts of collagens produced in different conditions and to investigate the proposed hypothesis in this study. Sections from articular cartilage of a pig joint were used as positive controls for assessing primary Col1 and Col2 antibodies by doing double immunofluorescence staining on the same section. Col2 staining appeared from superficial to calcified zones (all cartilage regions) that is normal for Col2 presence in the articular cartilage (Figure 4A) [33,34]. Additionally, staining of Col1 was present in the superficial zone ECM, while some staining in the calcified zone and portions of the deep zone seemed to be pericellular ECM or cellular (Figure 4B) [34,35,36]. Although both antibodies stained the subchondral bone region, negative control sections stained the same area with slightly stronger staining for Col1 in the negative control section (Figure 4C,D). Therefore, in the positive control section, positive staining was observed at the expected regions of Col1 and Col2, but not at those regions in the negative control section, confirming that the selected primary antibodies were effective with the staining protocol (Figure 4).

**Figure 5:**
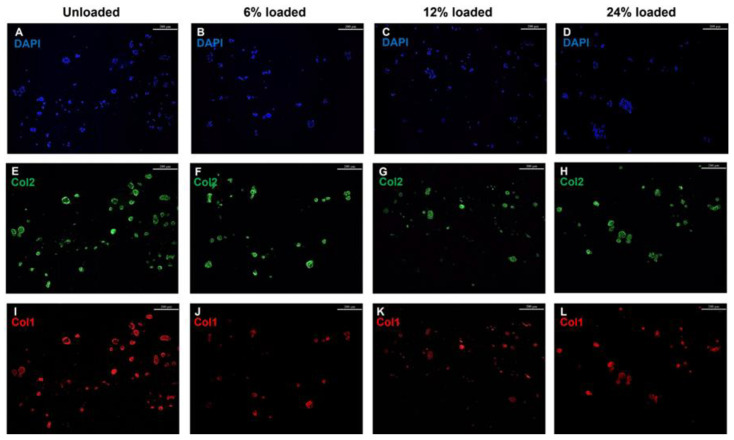
(**A**–**D**) Staining with DAPI of different groups showed that cell populations seemed to be lower in groups 12% and 24% loaded groups. (**E**–**H**) Col2 immunofluorescence staining confirmed chondrogenic differentiation of most of the cells for all the groups. (**I**–**L**) Col1 immunofluorescence staining showed its deposition from most of the cells in all groups suggesting that cells were differentiated to fibrochondrocytes (scale bars = 200 µm).

**Figure 6:**
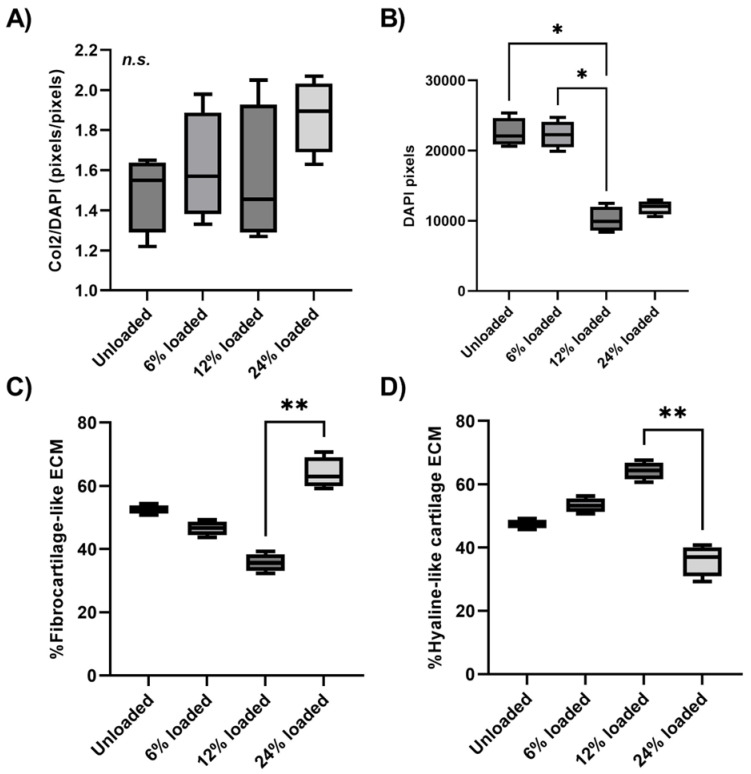
Quantitative results showed all groups seemed to have similar (**A**) Col2/DAPI quantities, but (**B**) DAPI pixels tended lower in 12% and 24% loaded groups. Additionally, (**C**) %hyaline-like cartilage and (**D**) %fibrocartilage-like ECM tended to be lowest and highest, respectively, in 12% loaded group. The bar graphs are showing median and interquartile range (n = 4 for each group, n.s. = not significant, * *p*  <  0.05, and ** *p*  <  0.01).

Immunofluorescence-stained sections from different groups visualized deposition of Col1 and Col2 from the impregnated cells (Figure 5). Positive Col2 areas in which Col1 was stained positive too (Col1-positive in Col2-positive pixels) were considered as fibrocartilage-like ECM, while Col2 was positive, but Col1 was negative (Col1-negative but Col2-positive pixels) were considered as hyaline-like cartilage ECM. Col2 deposition could be seen from most of the cells both in loaded and unloaded conditions confirming the chondrogenic differentiation of the cells (Figure 5E–H). Col1 staining was also positive for most of the cells suggesting that most of the ATDC5 cells were chondrogenically differentiated to fibrochondrocytes (Figure 5I–L). The unloaded and 6% loaded groups appeared to have higher Col2 staining across the sections than the 12% and 24% loaded groups (Figure 5E–H). However, DAPI staining suggested that number of the cells were also observed higher in the unloaded and 6% loaded groups (Figure 5A,B). Hence, higher Col2 staining would not necessarily guarantee a higher Col2 per cell population of the groups. However, a quantitative ratio of Col2/DAPI could be representative for Col2 deposition per cell population. Col1 deposition appeared to be present in all groups (Figure 5I–L), but the 12% loaded group seemed to have a qualitatively lower amount of Col1 deposition (Figure 5K). As a result, the ECM matrix of this group could be more composed of a hyaline-like cartilage matrix than the fibrocartilage-like one. Although, staining of the sections revealed that most cells deposited Col1 and Col2 concurrently, but areas for Col1- and Col2-positive pixels varied between the groups suggesting that amount of hyaline-like cartilage and fibrocartilage-like ECM could vary too. Therefore, quantitation was performed on the images to determine how much hyaline-like cartilage and fibrocartilage-like ECM were produced within different groups (Figure 6).

Mask images generated using Adobe Photoshop provided a clearer visualization of the differences between Col2 and also Col1 positive in Col2 positive areas, as well as DAPI pixels, which were used to quantitate the parameters discussed in the methods section. Based on the masked images from different groups, not all areas of positive Col2 necessarily are positive for Col1, and as a result, less Col1 pixels are present within the positive Col2 regions (Figure S1). In order to find how much Col2 is produced proportional to the cell population of each group, positive Col2 pixels were quantitated and normalized by DAPI pixels of the same section. According to the quantitation results, the levels of Col2/DAPI were similar across all groups with values of 1.49 ± 0.19, 1.61 ± 0.27, and 1.56 ± 0.35 for the unloaded, 6%, and 12% loaded groups, respectively (Figure 6A). The value seemed to be slightly higher in the 24% strain loading condition, at 1.87 ± 0.18, 25% higher than in unloaded condition; however, no significant differences were observed between the groups (Figure 6A). The quantitation results for DAPI pixels confirmed the qualitative observation from the images that less cells appeared to be present in the 12% and 24% loaded groups (Figure 6B). For the 12% and 24% loaded groups, respectively, DAPI pixels were 9394 ± 927 and 11915 ± 974, which tended to be lower than other groups without any significant differences (Figure 6B). Based on the Col1 and Col2 masked images, %fibrocartilage-like ECM deposition was quantitated for the groups (Col1-positive in Col2-positive pixels), and this ratio was 52.6 ± 1.4% and 46.6 ± 2.3% for the unloaded and 6% loaded groups, respectively (Figure 6C). In the 12% loaded group, the % fibrocartilage-like ECM seemed to decrease to 35.7 ± 2.8%. This measurement increased to 64.04 ± 4.9% for the 24% loaded group, and a statistical significance was only found between 12% and 24% loaded groups (Figure 6C).

Quantitation of %hyaline-like cartilage ECM would show an opposite trend as in the %fibrocartilage-like ECM since these measurements were basically complementary percentages. Hence, quantitation of %hyaline-like cartilage ECM (Col1-negative but Col2-positive pixels) confirmed that hyaline-like cartilage ECM seemed to be deposited higher in the 12% loaded group compared to the unloaded, 6% loaded, and also significantly higher than the 24% loaded groups (Figure 6D). Accordingly, this group had a 64.3 ± 2.3% hyaline-like cartilage ECM production, which tended to be 36% higher than the unloaded group. The 24% loaded group had quantitatively less deposition of hyaline-like cartilage ECM (at a level of 36.0 ± 4.9%) than the 12% loaded group, but it appeared to be at a close range as the unloaded and 6% loaded groups. As a conclusion, hyaline-like cartilage ECM production seemed to be highest in the 12% loaded group, with 1.36- and 1.79-fold higher than the unloaded and 24% loaded conditions, respectively (Figure 6D).

## 3. Discussion

It is essential to distinguish between hyaline cartilage vs. fibrocartilage ECM formed within the fabricated constructs to achieve a desirable tissue engineered articular cartilage. Cells within the implanted constructs may produce Col1 and/or Col2 in response to mechanical forces of the joints contributing to production of hyaline cartilage and/or fibrocartilage ECM [12,37]. The application of bioreactor systems helps us to understand cells responses to applied mechanical forces in mimicry of joint conditions where mechanical forces existing in the joints can be simulated [38,39]. Although GAG and Col2 productions were reported by several studies [13,14], the number of studies examining Col1 production is limited. However, increased Col1 production was seen in hydrogel constructs subjected to mechanical loading and in vivo [18,19,20]. Hence, in this study, it was hypothesized that compressive forces stimulate fibrocartilage development by increasing production of Col1.

This study was unique in terms of evaluating production of cartilage ECM of both hyaline cartilage and fibrocartilage in response to different compressive strains. To this aim, varying strains were examined on hydrogel constructs as inspired by the fact that articular cartilage of the joint deforms to various levels of thickness during various joint activities in vivo [27]. The 3D-bioprinted hydrogel constructs were mechanically compressed for 5 days for 3 h/day, and macroscopic observation of the loaded constructs showed that compression could cause damage to the constructs especially at 12% and 24% compressive strain conditions. According to these observations, longer loading periods may cause substantial damage, especially at 24% compression. Additionally, necessary precautions must be taken if longer periods of loading are intended to be examined by 6% and 12% loading conditions. Enhancing mechanical properties of the 3D-bioprinted hydrogels such as by increasing concentration of alginate hydrogel could help in protecting the loaded constructs. Fabrication and utilization of more robust constructs in future studies such as using natural/synthetic hybrid constructs is another way to reduce the degree of damage during loading [21].

Another consequence of extending the loading period would be the loss of the impregnated cells due to damage and degradation caused by compression. Additionally, cells might die under high compressive strains. This fact was confirmed by quantitating DAPI pixels revealing cell populations in different conditions. The unloaded and 6% loaded constructs had similar DAPI pixels, but this quantity was tended lower (about 0.5-fold) for the 12% and 24% loaded constructs. Since studies reported that cells survived different compression forces in vitro, the cell population might be diminished due to degradation-induced cell movement out of constructs [40,41].

Results from DMMB and hydroxyproline assays revealed production of GAGs and collagens as cartilaginous ECM molecules within the unloaded and loaded constructs confirming the differentiation of the impregnated cells. The amount of ECM produced within the constructs was not significantly changed by any loading condition. The presence of low ECM produced might also speed up degradation of alginate under high strain compression, since there was not enough ECM produced to fill the space within the hydrogel matrix and provide a support against degradation of the hydrogel matrix [42]. Low measured ECM at 12% strain could be associated with ECM and cell loss as it was shown by DAPI that less cells were present within the constructs of higher compressions. It was also reported by Kisiday et al. that dynamic stimulation with a similar strain caused higher ECM loss into the surrounding medium compared with unloaded hydrogels [43]. Regardless, it is highly recommended that ECM components as well as cell population are measured within constructs and in the media of the bioreactor to calculate the total amount of ECM produced and total cell populations.

Chondrogenic differentiation of the ATDC5 cell lines was confirmed by histology and immunofluorescence staining assessments. In the first attempt, safranin O was stained on the sections, but no stained matrix was discernable since calcium alginate, which is negatively charged with sulfate groups, strongly stained positive with safranin O [44]. Next, Alcian blue staining was attempted on the frozen sections. Although hydrogel matrix was stained with blue color resulted from the Alcian blue staining, the GAGs surrounding single cells or clusters of cells were stained with a darker blue color. GAG deposition was seen in all groups, confirming that cells were differentiated in the unloaded and varying loaded conditions. Immunofluorescence staining also showed positive Col2 staining around the cells proving the chondrogenic differentiation of the ATDC5 cells. From the histology and immunofluorescence images, hyaline cartilage specific molecules, including GAGs and Col2, were seen only around the cells and alginate matrix could be seen between the cells without any GAGs and Col2 which deposited in those areas. It was reported in previous studies that hydrogels have nano-sized pores which prevent cells from proliferating and infiltrating newly formed ECM, inhibiting the regeneration of new tissues [16]. Using the ATDC5 cell line in this study may have also contributed to low cartilage ECM production, as it was suggested that hydrogel constructs could regenerate more cartilage matrix using stem cells or primary cells instead of cell lines [45,46]. Although using ATDC5 cells led to low accumulation of ECM, these cells were established to undergo chondrogenic differentiation as many previous studies used them as an excellent in vitro model for chondrogenesis [47]. Hence, our study provides useful preliminary results in understanding how the cells respond to various mechanical stimulations.

Immunofluorescence images from different groups were analyzed visually and quantitatively to test the proposed hypothesis that mechanical compression stimulates fibrocartilage-like ECM production in the hydrogel constructs by increasing Col1 deposition. According to the immunofluorescence images, most of the cells deposited both Col1 and Col2 inferring that the ATDC5 cells were differentiated to fibrochondrocytes. To determine the amount of both hyaline-like cartilage and fibrocartilage-like ECM, quantitation was performed considering variations in Col1 deposition in positive Col2 regions. Although Col1 and Col2 images of double stained sections were taken at the exact same region, there was still misalignment of the images. To align the images, manual adjustments were carried out after overlaying on Photoshop software, which was helpful. The results of immunostaining and its quantitation revealed similar Col2/DAPI for all groups, although it seemed slightly higher in 24% loaded group. A similar results were also reported before with chondrocytes [48,49]; however, various contradictory findings in the literature suggest that the mechano-response of genes may depend a lot on compression conditions and hydrogel composition [50]. Quantitation of DAPI staining revealed a decrease in cell population at higher levels of compressive strain. This finding appears to contradict previous studies which reported an increase in cell proliferation when hydrogels are subjected to dynamic culture [51]. It is possible that the observed reduction in cell population in this study was a result of cells migrating out of the hydrogel structure. Such cell loss may occur due to fluid-mediated transport through the pores of the strands and porous networks within the hydrogels [52]. Based on our Col1 vs. Col2 quantitation, %fibrocartilage-like ECM produced in the 12% loaded constructs, which represented the compressive strain in the joints during normal walking [26], seemed to be decreased compared to the unloaded condition. This finding contradicts the proposed hypothesis in this study; however, this change was not statistically significant. The existence of few studies comparing Col1 and Col2 deposition concurrently at a physiological strain confirms this finding of the our study [40,53]. As a result of 12% strain dynamic compression, fluid flow could help transport nutrients and cytokines, leading to an increase in chondrogenesis and chondrocyte matrix composition [54]. Higher compressive strains exist in vivo that can occur during joint movements; thus, 24% strain was examined, for example. Compared to the other groups, 24% strain tended to have an increase in %fibrocartilage-like ECM production with a significant increase compared to the 12% loaded group. This finding confirmed the hypothesis of fibrocartilage-like ECM formation in response to increasing applied compressive strain. Two in vitro compression studies in the literature reported similar results for Col1 and Col2 production within hydrogels under high strain compressions. Hunter et al. [48] and Wang et al. [55] both reported elevation in Col1 production compared to Col2 while the chondrocyte incorporated hydrogels were compressed by high magnitudes of strains above 20%. However, the reason for that finding was uncertain, and yet, the intracellular mechanism governing force-sensing events of the chondrocytes and their mechanoresponses are not well-understood. ECM signals interact mechanically, biochemically, and fluid-dynamically throughout, making it challenging to determine how specific stimuli affect chondrocyte metabolism [56,57,58]. Finding the pathways regulating Col1 production and blocking them will help in producing less Col1 and fibrocartilage ECM at all compressive strains including high strains such as 24%.

## 4. Materials and Methods

### 4.1. Cell Culture

A murine chondrogenic cell line, ATDC5, as a well-established in vitro model for chondrogenic processes, was used in this study [47,59]. Cells were obtained from the RIKEN cell bank (Tsukuba, Japan). Frozen cells were thawed and cultured in a Petri dish containing Dulbecco’s modified Eagle’s medium/Ham’s nutrient mixture F12 (DMEM/F12, Sigma, D8900, St. Louis, MO, USA) supplemented with 5% fetal bovine serum (FBS, Gibco, USA) and 1% penicillin/streptomycin (Sigma, USA) (complete media). The culture media were changed every other day and the cells were incubated at 37 °C and 5% CO_2_. Cells were washed in about 90% confluency with phosphate-buffered saline (PBS, Sigma, P38135) and harvested after incubation for 5 min with 0.25% trypsin (Hyclone, Logan, UT, USA). The cell pellet was collected and suspended in a 175 T-flask containing complete media. Cultured cells were passed in more T-flasks until the required population of cells was obtained for 3D-bioprinting.

### 4.2. D-Bioprinting and Chondrogenic Culture of Hydrogel Constructs

Cylindrical shape hydrogel constructs were fabricated using a 3D-bioprinting technique as previously described [22]. Briefly, 6-well plates were coated with 0.1 mL of 0.1% (*w*/*v*) polyethyleneimine (PEI, Alfa Aesar, Mw: 60,000, Ward Hill, MA, USA) in PBS and incubated overnight at 37 °C to enhance printability of the alginate strands. The following day, medium viscosity alginate (Sigma-Aldrich, A2033) was dissolved in Stemline Keratinocyte Medium II-Calcium free (Sigma-Aldrich, S0196) and mixed with cell suspension in a 3:7 ratio (cell suspension volume to alginate volume) to achieve final alginate concentration of 2.5% and cell density of 5 × 10^6^ cells/mL. Cell-alginate suspension was transferred into a printing syringe with a 200 μm conical needle and mounted on a 3D-bioplotter machine (Envisiontec, Gladbeck, Germany). The PEI of the 6-well plates was replaced with sterilized 50 mM calcium chloride (CaCl2, Sigma-Aldrich) including 0.1% PEI, and cylindrical shape hydrogel constructs with 8 mm in diameter and height of 1.2 mm were fabricated by printing the cell-alginate suspension into 6-well plates. The fabricated constructs were transferred into a 6-well plate with DMED-F12 media and washed twice with media to remove excess CaCl_2_. Afterward, the constructs were transferred into a new 6-well plate with differentiation media, which contained complete media supplemented with 1% insulin-transferrin-selenium (ITS, Sigma, I3146) and 1% ascorbate-2-phosphate (Sigma, A8960). To initiate chondrogenic differentiation, the 3D-bioprinted constructs were cultured in the differentiation media for 10 days, and the media were changed every other day.

### 4.3. Dynamic Culture of Hydrogel Constructs

**Figure 7:**
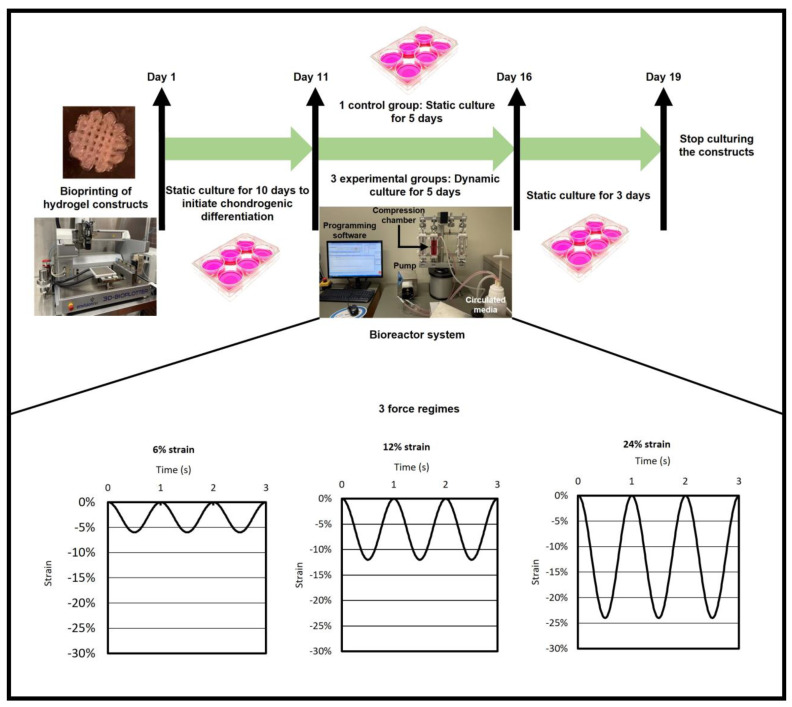
Overview of experimental design: Hydrogel constructs were fabricated using a 3D-bioplotter and cultured for 10 days. Afterward, they were either cultured in an unloaded condition or subjected to compression using a biodynamic machine. The three different compressive strains used are shown by the strain-time figures.

The cultured constructs were subjected to dynamic compression experiments to test the hypothesis that compressive force application stimulates fibrocartilage ECM production. To this end, compression experiments were carried out using a bioreactor (ElectroForce BioDynamic 5200, TA Instruments, Eden Prairie, MN, USA). The components of the bioreactor including the chamber, reservoir, and tubes were autoclaved and then assembled inside a biosafety cabinet. The chamber had two platens that were connected to two vertical arms. The chamber was also connected to a reservoir containing 300 mL of complete media using tubes. Constructs were placed on the lower platen of the chamber and the cleared doors were placed and secured to the front and back of the chamber. The chamber and its reservoir were transferred and mounted on the biodynamic machine afterward. The reservoir was placed and kept inside a 37 °C water bath during compression of the constructs and the media were circulated into the chamber using a peristaltic pump. Three sets of compression experiments were carried out with different strain magnitudes including 6%, 12%, and 24% to mimic various joint activities. In each experiment, four constructs were used with the same sinusoidal compression regime operating at 1 Hz for 5 days and 3 h/day except that the compressive strain varied for each compression experiment, as explained above. The compression regimes were programmed and controlled using WinTest software version 7.2 (Bose Corporation, Electroforce Systems Group). The constructs were transferred back into the 6-well plates after finishing compression on each day, and then, were cultured in a fresh differentiation media until the next day. After finishing the 5 days of compression experiments, the constructs were cultured for three more days inside the 6-well plates. A group of unloaded constructs was also cultured in static condition inside a 6-well plate as a control group for the same period as the loaded constructs were cultured. The whole experiment procedure is schematized in Figure 7.

### 4.4. Post-Culture Analyses

After three days following compression, the constructs from loaded and unloaded groups were cut in half. One half of each construct was assigned to be used for histology and immunofluorescence staining analyses, and the remaining half constructs were cut in half once more (two quarters of each construct), weighed using a scale, and stored in microtubes at −80 °C freezer for biochemical analyses.

The half-cut constructs were washed 2 times in Tris-buffered saline (TBS) containing 10 mM CaCl_2_ and fixed overnight at 4 °C using 4% Paraformaldehyde (PFA) mixed in TBS containing 10 mM CaCl_2_. Fixed samples were cryoprotected for 3.5 h on a shaker using 30% sucrose/Optimal Cutting Temperature (OCT, Tissue-Tek) (1:1) solution containing 10 mM CaCl_2_. In an ice bath, the samples were embedded inside a mold containing OCT solution. The OCT blocks were frozen and stored inside a −80 °C freezer.

#### 4.4.1. Histological Analysis

The OCT blocks of the half constructs were sectioned to 10 µm thickness using a cryotome (Fischer Scientific, Waltham, MA, USA). The sections were mounted on superfrost slides and stored in a −20 °C freezer until we used them for histological and immunofluorescence staining.

Alcian blue staining was used to visualize deposition of GAGs. To this end, frozen sections were first washed with 70% ethanol in 3% acetic acid solution for 15 min. Then, the slides were stained with 0.25% Alcian blue in 3% acetic acid solution for 4 h followed by destaining with graded ethanol solutions in 3% acetic acid over 3 h (50%, 75%, and 100% ethanol). The destained slides were washed with xylene, permount mounting medium was added on the slides, and images were taken at different magnifications using light microscopy (Nikon, Eclipse E600, SPOT Insight™ Camera, Melville, NY, USA).

#### 4.4.2. Digestion of Hydrogels

Samples assigned for biochemical assays (1 quarter of each construct) were removed from the freezer and thawed at room temperature. A protocol from the literature for dissolution and digestion of hydrogels was adopted and adjusted for use with our hydrogel constructs [60]. A dissolution solution (pH 7.5) containing 90 mM NaCl (Sigma-Aldrich), 100 mM sodium citrate (Sigma-Aldrich), 30 mM EDTA (Sigma-Aldrich), 0.1 mM Tris HCl (Sigma-Aldrich), 0.2 mM CaCl_2_ (Sigma-Aldrich), 0.2 mg/mL proteinase K (Roche, Mannheim, Germany) was prepared and added to the tubes containing hydrogel samples. The dissolution solution was added to the tubes with a ratio of 1:10 (µg of hydrogel:µL of dissolution solution), and pipetted to dissolve the alginate hydrogel and obtain the cells and surrounding ECM released. Additionally, presence of proteinase K in the solution caused the digestion of cells and their ECM. The tubes were incubated at 55 °C overnight with proteinase K to digest the cells. Digested solutions were centrifuged for 3 min at 10,000 rpm and the supernatants were used for two separate analyses of dimethylmethylene blue (DMMB) and hydroxyproline (HP) assays.

#### 4.4.3. Determination of GAG Production

Several protocols were combined and modified to generate a DMMB assay for our study [60,61,62,63]. A mixture was prepared by dissolving 21 mg of DMMB (Sigma, 341088) in 5 mL of absolute ethanol, followed by the addition of 2 g of sodium formate (Sigma). Then, the solution was mixed with 800 mL of double distilled water. Formic acid (95%, Sigma) was added dropwise to the solution until pH was 1.5, and then, distilled water was added to make 1L of final solution.

The process involved transferring 50 µL of centrifuged and digested samples into a 96-well plate. To this, 250 µL of DMMB was added to each well, and the contents were mixed by pipetting up and down. After incubation at room temperature for 1 h, a spectrophotometric plate reader (Thermo Fisher) was used to measure optical density (OD) of each well at 525 nm. A linear standard curve was also generated using various known concentrations of type A chondroitin sulfate (from bovine trachea, Sigma-Aldrich), and the GAG content of the hydrogel samples was extrapolated from the standard curve.

#### 4.4.4. Determination of Total Collagen Production

Hydroxyproline assay is a stablished protocol to determine the amount of total collagen produced from the cells. This assay was performed on our samples according to adjustments from the manufacturer’s suggestions and available protocols in the literature [64,65]. The samples used for the hydroxyproline assay were also prepared using the dissolution protocol above using a kit purchased from Abcam (ab222941). The samples were vortexed, and then hydrolyzed in a microtube at 100 °C for 4 h with the same amount of 10 N HCl used for all samples (1:1 for sample volume to HCl volume ratio). The hydrolysate samples were centrifuged at 10,000 rpm for 3 min to remove precipitates, and 10 μL of each sample’s supernatant was added into a 96-well plate. The samples were evaporated until dry by heating at 65 °C for 2 h. To oxidize the hydrolysate samples, 100 μL of chloramine-T buffer (6 μL of Chloramine T Concentrate mixed in 94 μL Oxidation Buffer) was added to each well and incubated at room temperature for 5 min. As a final step, the wells were incubated with 100 μL of dimethylaminobenzaldehyde (DMAB) solution (DMAB concentrate mixed in a developer solution (1:1)) for 90 min at 60°C inside an oven. After removing the 96-well plate from the heat source, the absorbance of the samples was read at 560 nm in endpoint mode. The hydroxyproline content of the samples was obtained using the linear standard curve for standard hydroxyproline solutions (0–50 µg/mL). The collagen content of the constructs was calculated by converting hydroxyproline content to collagen content using 7.6 as a conversion factor [66].

#### 4.4.5. Analysis and Quantitation of Immunofluorescence Staining

Immunofluorescence staining was carried out on the section to detect deposition of COL1 and COL2 from the cells. The frozen slides were rehydrated using a series of ethanol and xylene, and then, baked in an oven at 55 °C for 15 min. The sections were permeabilized with 0.5% Triton X-100 in PBS (PBST) for 5 min and digested by 0.1% trypsin (MP biomedicals, 153571, Irvine, CA, USA) in PBST for 15 min at 37 °C. The slides were then washed with PBST for 5 min followed by incubating with 0.5% hyaluronidase (Worthington, Lakewood, NJ, USA) in PBST (0.005g/mL) for antigen retrieval for 15 min at 37 °C. Afterwards, the sections were blocked for non-specific bindings using blocking buffer containing 4% natural goat serum (Sigma, G9023) and 2% natural sheep serum (Sigma, S3772) in PBS-T for 1 h at room temperature. The blocked sections were then incubated with blocking buffer containing primary antibodies of both anti-Col1 (1:100, BioRad, 2150-1410, Hercules, CA, USA) and anti-Col2 (1:100, DSHB, II-II6B, Iowa City, IA, USA) overnight at 4 °C. Using this protocol, the sections were double-immunostained with primary antibodies, so that Col1 and Col2 were detected on the same exact section. The next day, the sections were washed twice with PBST each time for 5 min, and secondary antibodies including goat anti-rabbit IgG-594 (1:1000, Thermo Fisher, applicable for primary anti-Col1) and goat anti-mouse IgG-488 (1:1000, Thermo Fisher, applicable for primary anti-Col2) diluted in blocking buffer were applied on the sections for 3 h in the dark and at room temperature. The sections were washed twice with PBST (5 min each step), and then mounted with mounting medium containing DAPI (Vectashield, Vector Laboratories, Burlingame, CA, USA). Afterwards, images were taken from different regions of the sections using a fluorescence microscope (Nikon, Eclipse E600, SPOT Insight™ Camera, USA). As a result, Col1, Col2, and DAPI were visualized in red, green, and blue, respectively. Adobe Photoshop software (Adobe systems Inc., version 13.0, San Jose, CA, USA) was utilized to quantitate the total number of pixels for positive DAPI, Col2, and then, calculate DAPI pixel intensity representing cell population and Col2/DAPI representing deposited Col2 per cell for the different groups. Then, Col1 and Col2 images were opened as layers in Photoshop, and Col2 images were used to generate masks of positive Col2 areas. These masks were applied on Col1 images to generate masks for positive Col1 area within positive Col2 area, and from there, number of positive red pixels were calculated within these mask areas (Col1-positive in Col2-positive pixels). Afterwards, %Col1/Col2 could be calculated for each image, and this ratio represented quantitated percentage area of formed fibrocartilage-like ECM to total formed cartilage ECM (=%fibrocartilage-like ECM) for each image. By subtracting positive Col1 within the positive Col2 area from the total Col2 area (Col1-negative but Col2-positive pixels), % (1 − Col1/Col2) could be calculated for each image that represented percentage area of the formed hyaline-like cartilage ECM to the total formed cartilage ECM (=%hyaline-like cartilage ECM). It is worth also emphasizing that at least 6 field of views (6–8) per sample were included for quantitation.

### 4.5. Terminology

Whitin this manuscript, different experimental groups are defined as unloaded, 6% loaded, 12% loaded, and 24% loaded. Additionally, using the terminology introduced in the above section, %fibrocartilage-like ECM and %hyaline-like cartilage ECM are used to refer quantitative measurements for %Col1/Col2 and % (1 − Col1/Col2), respectively.

### 4.6. Statistical Analysis

Statistical analyses were performed with GraphPad Prism software package version 9.4.1. As all groups had small sample sizes, non-parametric statistical Kruskal–Wallis one-way analysis of variance (ANOVA) followed by Dunn’s multiple comparisons test was used. Quantitative results are presented the mean value with the corresponding standard deviation within the context, while the figures depict the median and the interquartile range (IQR), and statistical significance was accepted for *p* values of less than 0.05.

## 5. Conclusions

Our study examined the amount and type of cartilaginous ECM that could be produced by applying different compressive forces on 3D-bioprinted hydrogel constructs. In both unloaded and loaded conditions, chondrogenic differentiation of impregnated cells were observed by means of histological, immunofluorescence, and biochemical methods. In addition to causing substantial damage to the 3D-bioprinted constructs, higher compressive strains decreased the cell population within the constructs. The results showed that magnitude of applied compressive strain affected the amount of hyaline-like cartilage vs. fibrocartilage-like ECM produced. A 6% strain compression seemed not to change the composition of the ECM as compared to the unloaded constructs for total collagen, GAGs, Col2, and hyaline-like cartilage ECM production. By contrast, the 12% compressive strain tended to generate more hyaline-like cartilage ECM than other groups. However, further studies are required to improve the total collagen and GAG production in this strain magnitude, since it tended to be the lowest among all groups. The substitution of primary cells or stem cells might be helpful in this endeavor. Compared to the other compression and unloaded groups, 24% strain tended to produce higher total collagen, GAGs, and Col2 within the constructs, but it also stimulated the cells to form more fibrocartilage-like ECM. Further enhancements such as blocking the mechanotransduction pathways regulating Col1 production would help to reduce Col1 deposition and produce more hyaline-like cartilage ECM under compressive forces.

## Data Availability

The data that support the findings of this study are available upon reasonable request from the corresponding author.

## Supplementary Materials

The following supporting information can be downloaded at: https://www.mdpi.com/article/10.3390/ijms24087410/s1.
