# Supplementary material for: Applied Compressive Strain Governs Hyaline-like Cartilage versus Fibrocartilage-like ECM Produced within Hydrogel Constructs
Source: Int J Mol Sci. 2023 Apr 18;24(8):7410. doi: 10.3390/ijms24087410 (PMC10138702; doi:10.3390/ijms24087410)
Supplement: Supplementary file 1 [file ijms-24-07410-s001.zip › ijms-2334431-supplementary.pdf]

Supplementary figure:

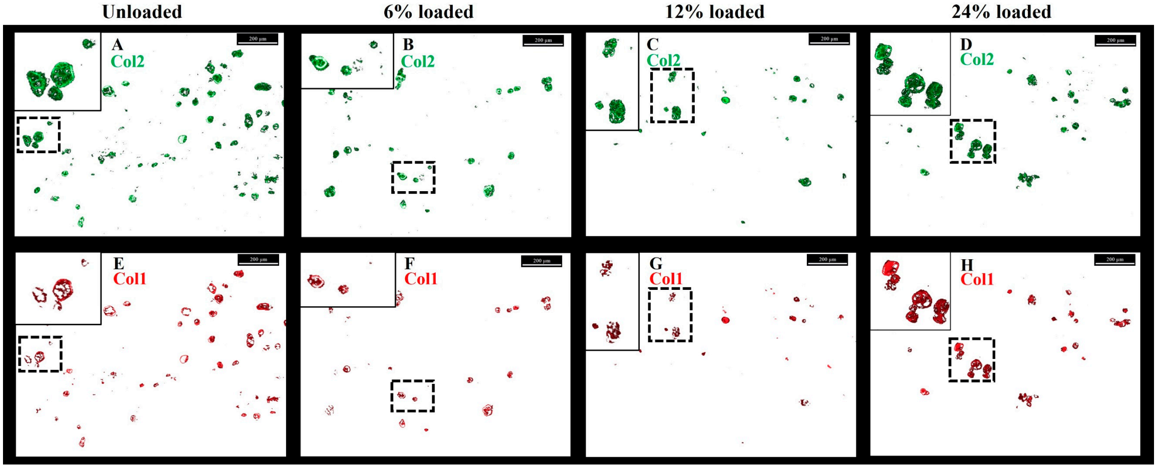

Figure S1: Masked immunofluorescence images of (E-H) Col1 and (A-D) Col2 showed less Col1 deposition in Col2 deposited areas of all groups. 24% loaded groups looked having more deposited Col1 compared to the other groups (Scale bars = 200 μm).
